# Lemon-derived nanovesicles achieve antioxidant and anti-inflammatory effects activating the AhR/Nrf2 signaling pathway

**DOI:** 10.1016/j.isci.2023.107041

**Published:** 2023-06-07

**Authors:** Ornella Urzì, Marco Cafora, Nima Rabienezhad Ganji, Vincenza Tinnirello, Roberta Gasparro, Samuele Raccosta, Mauro Manno, Anna Maria Corsale, Alice Conigliaro, Anna Pistocchi, Stefania Raimondo, Riccardo Alessandro

**Affiliations:** 1 Dipartimento di Biomedicina, Neuroscienze e Diagnostica Avanzata (Bi.N.D), sezione di Biologia e Genetica, Università degli Studi di Palermo, 90133 Palermo, Italy; 2 Dipartimento di Biotecnologie Mediche e Medicina Traslazionale, Università degli Studi di Milano, LITA, Via Fratelli Cervi 93, Segrate, 20090 Milano, Italy; 3 Istituto di Biofisica, Consiglio Nazionale delle Ricerche, 90146 Palermo, Italy; 4 Central Laboratory of Advanced Diagnosis and Biomedical Research (CLADIBIOR), AOUP Paolo Giaccone, Palermo, Italy; 5 Istituto per la Ricerca e l’Innovazione Biomedica (IRIB), Consiglio Nazionale delle Ricerche, 90146 Palermo, Italy

**Keywords:** Molecular biology, Immunology, Cell biology

## Abstract

In the last years, extracellular vesicles (EVs) from different plant matrices have been isolated and gained the interest of the scientific community for their intriguing biological properties. In this study, we isolated and characterized nanovesicles from lemon juice (LNVs) and evaluated their antioxidant effects. We tested LNV antioxidant activity using human dermal fibroblasts that were pre-treated with LNVs for 24 h and then stimulated with hydrogen peroxide (H_2_O_2_) and UVB irradiation. We found that LNV pre-treatment reduced ROS levels in fibroblasts stimulated with H_2_O_2_ and UVB. This reduction was associated with the activation of the AhR/Nrf2 signaling pathway, whose protein expression and nuclear localization was increased in fibroblasts treated with LNVs. By using zebrafish embryos as *in vivo* model, we confirmed the antioxidant effects of LNVs. We found that LNVs reduced ROS levels and neutrophil migration in zebrafish embryos stimulated with LPS.

## Highlights

• Nanovesicles from lemon juice (LNVs) were isolated and characterized
• LNVs have antioxidant effects on human dermal fibroblasts by reducing ROS levels
• LNVs activate the AhR/Nrf2 pathway in human dermal fibroblasts
• LNVs have anti-inflammatory and antioxidant effects in zebrafish embryos

## Graphical abstract

**Figure:**
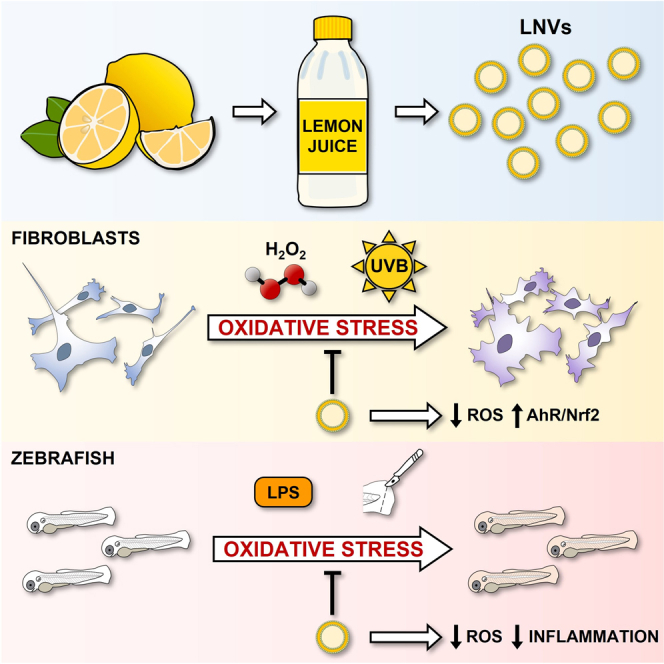

## Introduction

In the last decades, extracellular vesicles (EVs) have been emerging as mediators of cross-kingdom communication; being released from different organisms they represent a “common language,” acting as nanoscale packages of biological messages.^1^^,^^2^ EVs were extensively described in mammalians where they were first considered as “garbage bins;”^3^^,^^4^ however, since the study of Valadi et al.,^5^ which demonstrated the presence of functional RNAs inside EVs, they raised the interest of the scientific community and started to be investigated also in other kingdoms.

Plant-derived EVs (PDEVs) can be isolated from different edible fruits and vegetables, such as lemon,^6^^,^^7^ grapefruit,^8^ and tomato,^9^ and are stimulating interest in researchers thanks to their promising features. PDEVs showed several similarities to mammalian EVs in terms of size and morphology,^10^ their cargo includes lipids,^11^ proteins,^6^ nucleic acids,^12^ and metabolites.^7^^,^^13^ Many studies highlighted the biological properties of PDEVs, including anti-cancer,^6^^,^^14^ anti-inflammatory,^8^ and anti-oxidant activities.^15^

The oxidative stress is caused by the imbalance between the production of oxygen reactive species (ROS) and the cellular antioxidant response.^16^ ROS are normally produced as byproducts of oxygen metabolism; however, an excessive increase in their levels results in damage to essential molecules, such as DNA, lipids, and proteins.^17^ Oxidative stress is involved in the onset and progression of several pathological conditions, including cancer,^18^ diabetes,^19^ cardiovascular,^20^ and neurodegenerative diseases.^21^

The aryl hydrocarbon receptor (AhR) is a cytoplasmic transcription factor that acts as an environmental sensor^22^; following the binding with its ligands, which include metabolites and polyphenols,^23^ it translocates into the nucleus and activates the transcription of target genes.^22^ AhR nuclear translocation induces an anti-oxidant response through the activation of the nuclear factor-erythroid factor 2-related factor 2 (Nrf2).^24^ Nrf2 plays in turn a key role in the protection of cells against oxidative stress by activating the transcription of several antioxidant enzymes.^25^ Interestingly, natural compounds have proven to protect cells from oxidative stress by activating AhR/Nrf2 signaling pathway.^26^^,^^27^

PDEVs, as mentioned previously, possess interesting beneficial activities; it was found that EVs isolated from carrots counteracted oxidative stress. Carrot-EVs inhibit ROS production and upregulate the expression of Nrf-2, HO-1, and NQO-1, in cardiomyoblasts and neuroblastoma cells.^15^ Moreover, EVs from other plant matrixes, such as blueberries^28^ and strawberries^29^ showed anti-oxidant effects on endothelial and mesenchymal cells, respectively. Recently, we have demonstrated that nanovesicles isolated from the juice of *Citrus limon* (LNVs) exert anti-inflammatory effects on murine and human immune system cells through the inhibition of NF-κB/ERK1-2 pathways.^7^

Here, our studies focused on the investigation of their anti-oxidant activity by the use of *in vitro* and *in vivo* models.

## Results

### LNVs can be internalized by human dermal fibroblasts without affecting their viability

**Figure 1:**
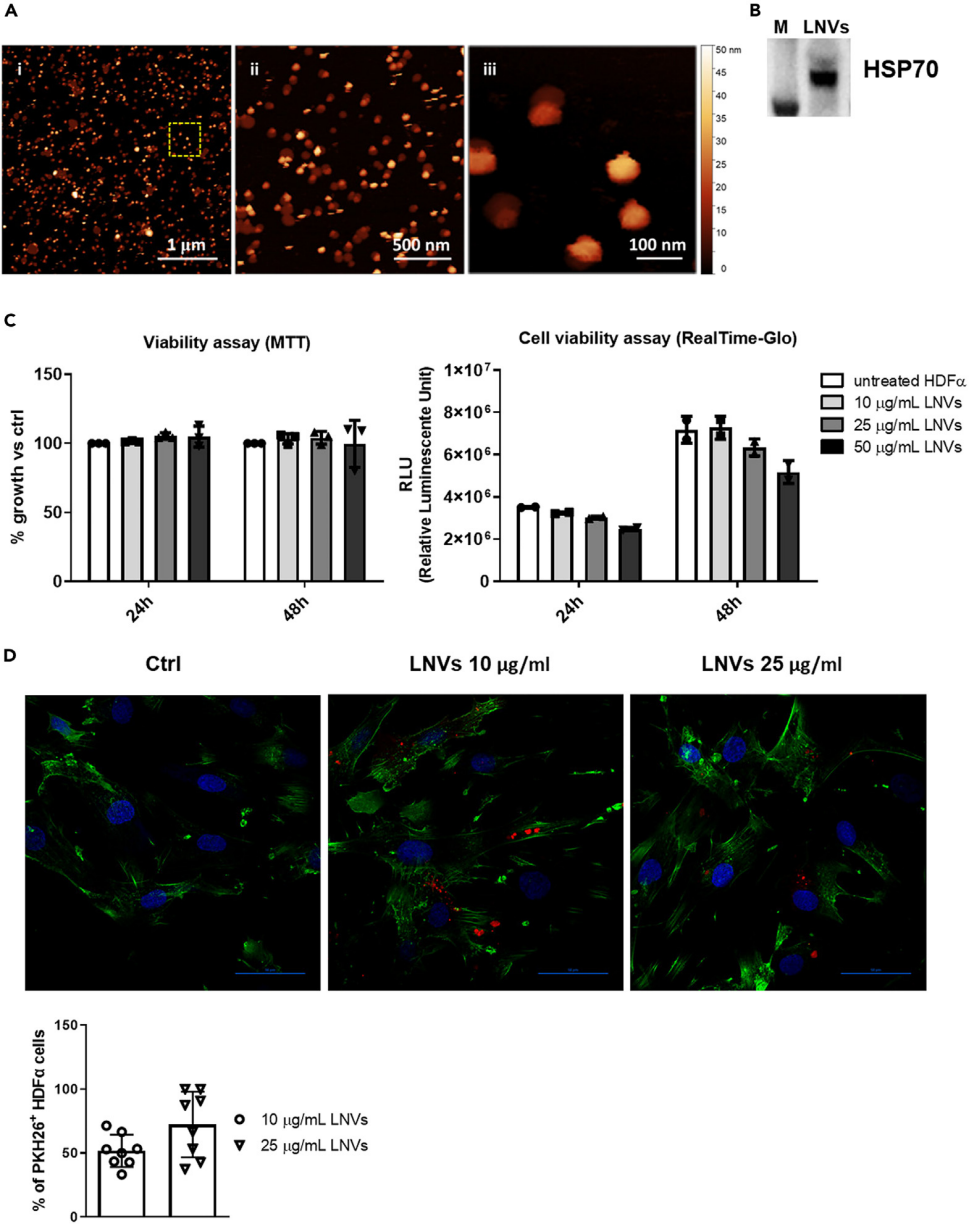
Characterization and uptake of LNVs by HDFα cells (A) Atomic force microscopy images of Citrus LNVs acquired in quantitative Imaging in liquid at different magnifications. Panel iii is a zoom of the marked area in panel i. (B) Western blot analysis of HSP70 in LNVs. (C, left panel) MTT assay (n = 3) and (C, right panel) Real-Time Glo (n = 2) of HDFα cell line treated with different doses of LNVs (10, 25, and 50 μg/mL) for 24 and 48h. Data are represented as mean ± SD. (D) LNVs internalization by HDFα cells, LNVs were stained with PKH26 (in red), actin with actin green (in green), and nuclei with Hoechst (in blue). Scale bars are 50 μm. The histogram reports the percentage of PKH26 positive cells. Data are represented as mean ± SD.

Once LNVs were isolated, they were characterized through atomic force microscope (AFM) and western blotting. AFM images showed a main population of vesicles with a size of about 80 nm wide (and vertical height of 30 nm), compatible with average size objects observed by dynamic light scattering in previous work.^6^ At the same time, images showed a second population of large and quite flat objects (about 10 nm high), likely associated with the collapse of vesicles, a casual effect that could be ascribed to their direct interaction with mica surface (Figure 1A). NTA analysis showed that LNVs possessed a mean size distribution of 65nm ± 2.7 nm and a mode of 48.4 nm ± 0.2 nm (Figure S1A). LNVs also presented the known EV biomarker HSP70 (Figures 1B and S1B). These results indicated that LNVs have canonical EV characteristics according to MISEV guidelines.^30^

To study the possible *in vitro* anti-oxidative effects of LNVs, we selected human dermal fibroblasts (HDFα cells). They represent the main cell type of the skin connective tissue and are exposed to extrinsic stimuli, such as ultraviolet (UV) irradiation and pollution, which could unbalance the redox state equilibrium.^31^ First, we analyzed the viability of HDFα cells following the treatment with increasing doses of LNVs through MTT and RealTime Glo assays. The nanovesicles intake did not affect HDFα cell viability at both 24 and 48 h (Figure 1C). The highest dose (50 μg/mL) slightly reduced cell viability thereby it was not selected for subsequent experiments (Figure 1C). We then labeled LNVs with PKH26 and incubated them with HDFα cells for 4 h at 37°C to ensure that HDFα cells were able to internalize them. Fluorescent microscopy analysis showed that LNVs were successfully internalized by human dermal fibroblasts and localized inside the cells, mainly in the perinuclear region (Figure 1D). We also performed the assay at 4°C; we observed that the LNV internalization was unpaired, thus confirming that nanovesicles uptake was mediated by a biologically active process (Figure S1C).

Overall, these data demonstrated that LNVs possessed canonical EVs features and interact with human dermal fibroblasts without affecting their viability.

### LNVs enhanced the functions of human dermal fibroblasts

**Figure 2:**
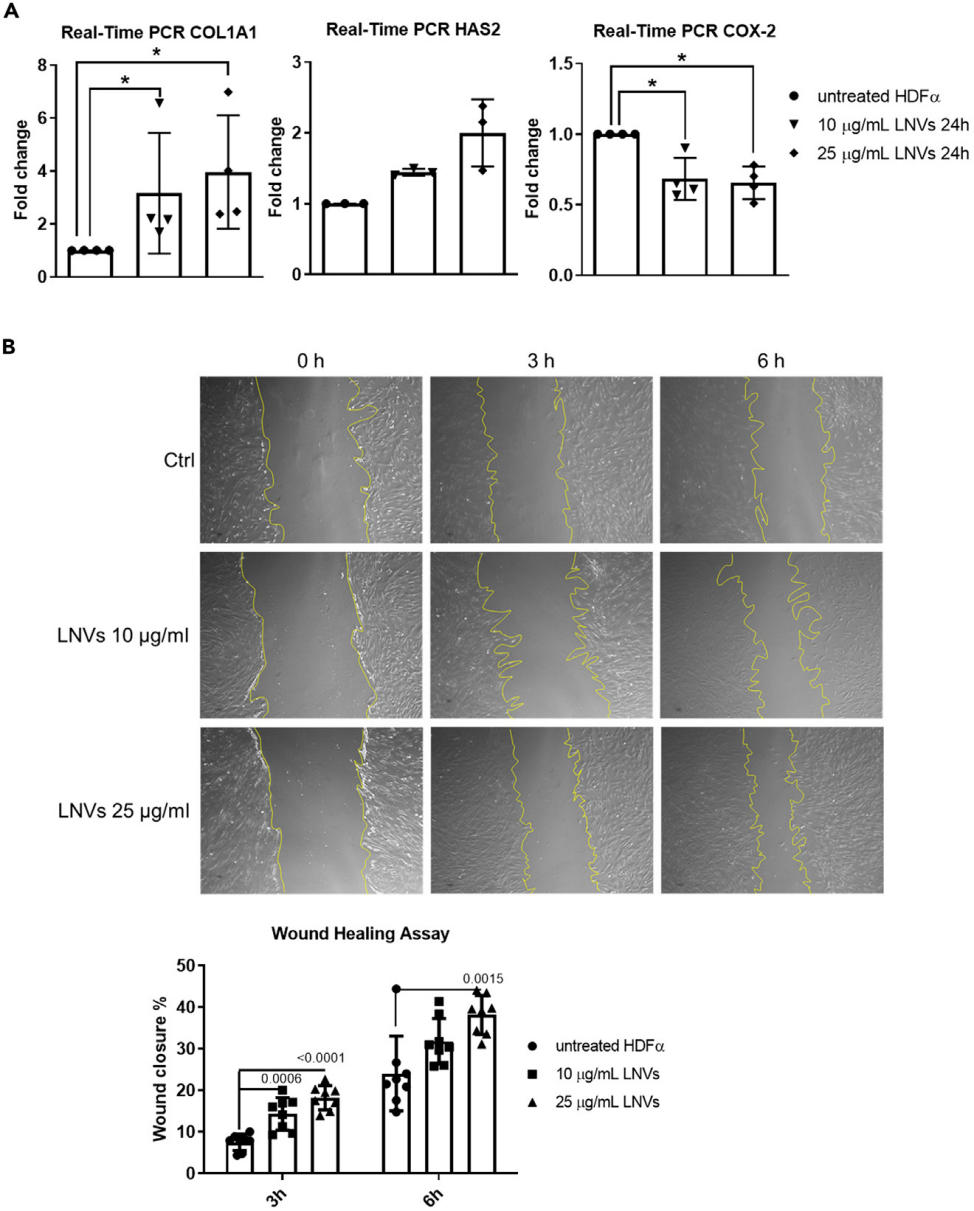
LNVs increased the production of ECM molecules and promoted wound healing of HDFα cells (A) RT PCR of *COL1α1* (n = 4), *HAS2* (n = 3), and *COX-2* (n = 4) mRNA levels in HDFα cell line treated with LNVs (10 and 25 μg/mL) for 24h. Data are represented as mean ± SD. Statistical significance was assessed by non-parametric Mann-Whitney test; ∗p < 0.05. (B) Wound healing assay of HDFα cell line pre-treated with LNVs (10 and 25 μg/mL) for 24h (n = 8). Then the scratch was made with a sterile 200 μL pipet tip and cells were observed at 0, 3, and 6 h. The percentage of wound closure was calculated as described in the STAR Methods section. Data are represented as mean ± SD. Statistical significance was assessed by unpaired Student’s *t* test (the normal data distribution were assessed by Shapiro-Wilk test test).

Once we ensured that LNVs can be internalized by HDFα cells and did not induce cytotoxicity, we investigated the effects of LNVs treatment on human dermal fibroblast functions. Dermal fibroblasts play a key role in the maintenance of skin homeostasis by producing the extracellular matrix (ECM).^32^ We found a significant increase of collagen (*COL1α1*) in HDFα cells treated for 24 h with LNVs and a trend of increase in hyaluronic acid synthase 2 (*HAS2*) (Figure 2A). At the same time, we observed a decrease in Cyclooxygenase-2 (*COX-2*) (Figure 2A), an enzyme involved in chronic inflammation, whose reduction has been previously correlated with an improved cutaneous wound healing.^33^ Human dermal fibroblasts also have a predominant role in wound repair and remodeling,^34^ for this reason, we analyzed the wound-healing capability of HDFα cells following the treatment with LNVs. As shown in Figure 2B, we observed a dose-dependent increase in the wound closure percentage when HDFα cells were treated for 3 and 6 h with 10 and 25 μg/mL of LNVs. These data suggested that LNVs could enhance ECM formation thereby favoring the wound repair process of human dermal fibroblasts.

### LNVs exert an antioxidant effect on HDFα by reducing ROS levels

**Figure 3:**
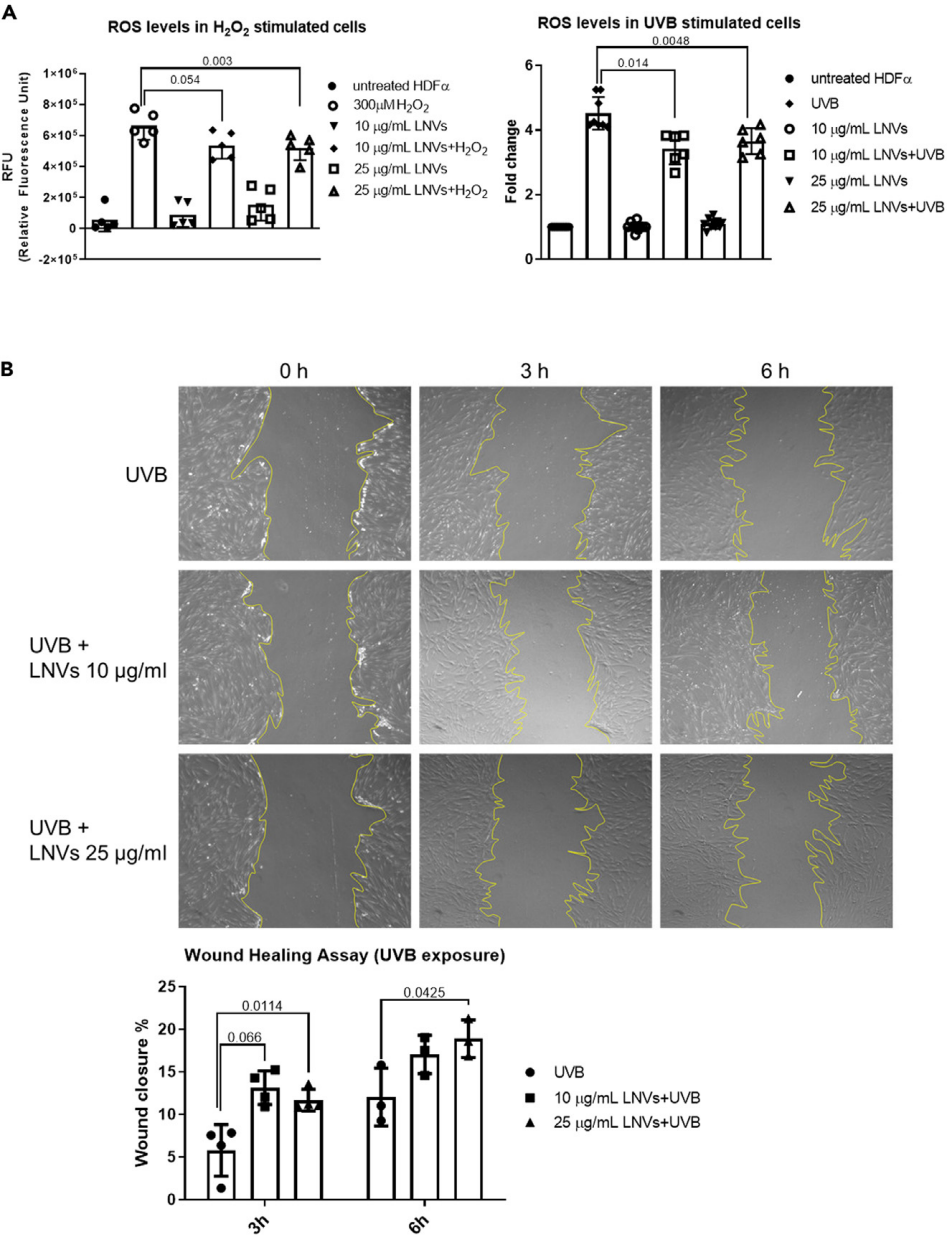
LNVs decreased the amount of ROS *in vitro* (A, left panel) ROS levels of HDFα cell line pre-treated with LNVs (10 and 25 μg/mL) for 24 h and then stimulated with H_2_O_2_ (n = 5). (A, right panel) ROS levels of HDFα cell line pre-treated with LNVs (10 and 25 μg/mL) for 24 h and then stimulated with UVB irradiation for 25 s (n = 6). Data are represented as mean ± SD. (B) Wound healing assay of HDFα cell line pre-treated with (10 and 25 μg/mL) for 24 h (n = 3–4). After doing the scratch with a sterile 200 μL pipet tip, the cells were irradiated with UVB for 25 s and observed at 0, 3, and 6 h. The percentage of wound closure was calculated as described in the STAR Methods section. Data are represented as mean ± SD. Statistical significance was assessed by unpaired Student’s *t* test (the normal data distribution were assessed by Shapiro-Wilk test test).

As mentioned previously, external stimuli can increase the production of ROS thus inducing skin aging, inflammation, and disease.^35^ To investigate the possible anti-oxidative effect of LNVs, we selected two known oxidative stress stimuli: hydrogen peroxide (H_2_O_2_)^36^ and UV irradiation.^37^ We pre-treated HDFα cells with 10 and 25 μg/mL of LNVs and then induced the oxidative stress by exposing the cells to H_2_O_2_ (300 μM) for 4 h or UVB irradiation (20 mJ/cm^2^) for 25 s. Then we evaluated ROS levels using the DCFDA probe and we found that both H_2_O_2_ and UVB irradiation (Figure 3A) were able to increase ROS levels in HDFα compared to the control condition, thus representing a valuable stimulus to induce oxidative stress. On the other hand, LNV pre-treatment counteracted the ROS increment caused by H_2_O_2_ treatment (Figure 3A, left panel) or UVB irradiation (Figure 3A, right panel).

Moreover, we investigated the pro-healing properties of LNVs in oxidative stress conditions. Firstly, we found that UVB irradiation inhibited wound closure, especially 6 h after the scratch generation (Figure S1D). Then, following 24 h of pre-treatment with LNVs (10 and 25 μg/mL), a scratch was generated and HDFα cells were exposed to UVB irradiation for 25 s. We found that the pre-treatment with 25 μg/mL of LNVs favor wound closure compared to untreated cells (Figure 3B), thus confirming the pro-regenerative effects of LNVs also in oxidative stress conditions.

These results indicated that LNVs were able to protect human dermal fibroblasts from oxidative stress by reducing the production of ROS.

### LNVs activate AhR/Nrf2 antioxidant pathway in human dermal fibroblasts

**Figure 4:**
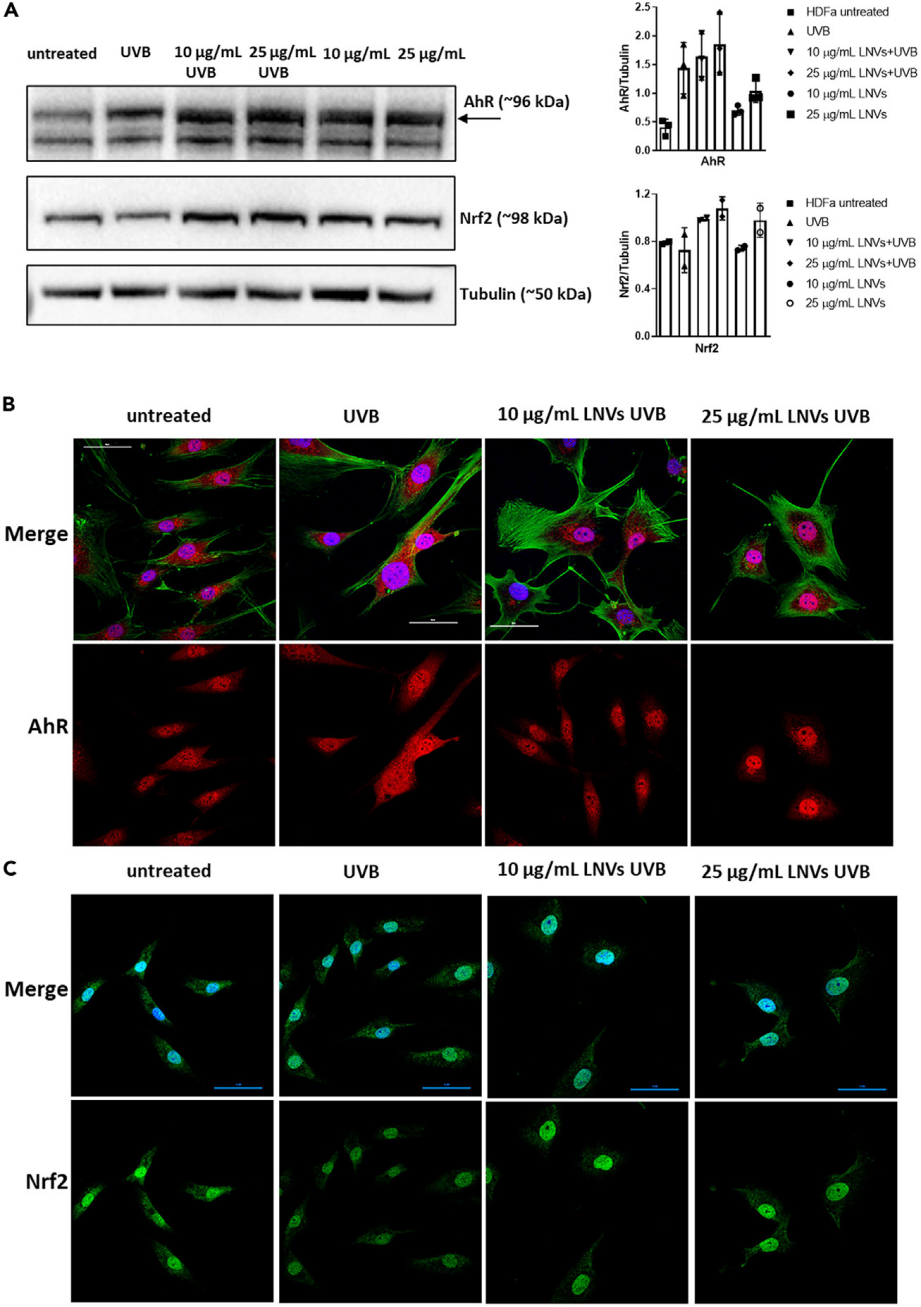
LNVs activate the AhR/Nrf2 signaling pathway (A) Western blot analysis of AhR (n = 3) and Nrf2 (n = 2) levels in HDFα cell line pre-treated with LNVs (10 and 25 μg/mL) for 24 h and then stimulated with UVB irradiation (20 mJ/cm^2^) for 25 s. Data are represented as mean ± SD. (B and C) Confocal analysis of AhR (B, red signal) and Nrf2 (C, green signal) levels in HDFα cell line untreated, or treated with LNVs (10 and 25 μg/mL) for 24h in the presence of UVB; actin was stained with actin green (in green), nuclei with Hoechst (in blue). Scale bar 50 μm.

Once we demonstrated that LNVs were able to reduce ROS levels *in vitro*, we evaluated the signaling pathway underlying the LNV-mediated antioxidant response. In particular, we focused on AhR/Nrf2 signaling pathway, since previous studies demonstrated that it can be activated in the anti-oxidant response.^38^^,^^39^ It is known that environmental stress, such as UVB irradiation, may activate AhR,^40^ but the presence of anti-oxidant agents can induce Nrf2 expression to counteract oxidative stress.^41^ In this scenario, we pre-treated cells with LNVs for 24 h and then induced oxidative stress using UVB irradiation to understand whether LNVs may act as antioxidant agents also in stress conditions. Interestingly, we observed that LNVs were able to upregulate the protein expression of AhR and NRF2 in human dermal fibroblasts stimulated with UVB irradiation (Figures 4A and S2). These results suggested that LNVs could activate AhR/Nrf2 signaling pathway under physiological conditions (without UVB irradiation) and this activation is maintained under oxidative stress conditions (UVB irradiation). Moreover, through confocal analysis, we observed an increase in the nuclear localization of AhR (Figure 4B) and Nrf2 (Figure 4C) in LNV-treated HDFα cells.

### LNVs possess a protective role against oxidative agents *in vivo*

To provide valuable insights into the prophylactic effects of LNVs administration *in vivo*, we took advantage of the zebrafish (*Danio rerio*) model. Zebrafish larvae at 48h post-fertilization (hpf) were treated for 24 h with LNVs dissolved in embryo medium E3 + PTU.

Different concentrations of LNVs were tested: 10, 25, and 50 μg/mL. As a control, embryos were incubated with the E3 medium + PTU without LNVs. All doses of LNVs were well tolerated, and the larvae did not show any gross morphological defects (Figure S1E). We decided to perform the following analyses using the dose of 25 μg/mL of LNVs.

**Figure 5:**
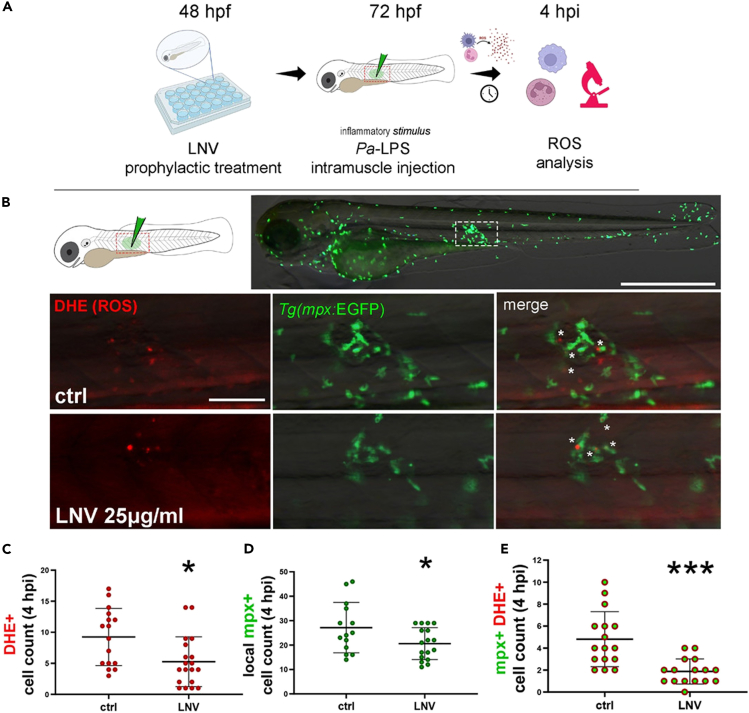
Antioxidant effects of prophylactic treatment with LNVs on innate immunity of zebrafish embryo 48 hpf embryos were prophylactically treated with 25 μg/mL of LNVs and then undergone *Pa-LPS*-induced (A–E) local inflammatory stimulus. (A) Schematic representation of LPS-induced stimulus model; (B) representative images at 4 hpi of neutrophils recruitment and ROS production (DHE) at the site of *Pa-*LPS intramuscular injection, in the whole embryo (upper) and the region of interest of the trunk (lower panels), in embryos pre-treated or not with LNVs; white asterisks indicate *mpx*^+^DHE^+^ cells; (C–E) quantitative analysis at 4 hpi of DHE^+^ cell count (C), *mpx*^+^ cell count (D) and *mpx*^+^DHE^+^ cell count (E) at the region of interest. Mean and SEM of at least two independent experiments are shown; dots represent cell count in a single embryo. Statistical significance was assessed by unpaired Student’s *t* test followed by Welch’s correction for C and D (the Gaussian data distribution were assessed by Kolmogorov-Smirnov normality test) or by non-parametric test (Mann-Whitney test) for E.

We then verified if the prophylactic administration of LNVs might improve the modulation of the innate immune system following an acute inflammatory stimulus done by treatment with lipopolysaccharide *Pa*-LPS. *Tg(mpx:GFP)* larvae at 72 hpf pre-treated with LNVs, were injected with *Pa*-LPS in the skeletal muscles of the trunk region to perform a localized inflammation (Figure 5A). Neutrophils activation and ROS production were measured 4 h post injection (hpi) (Figure 5B). Both cell producing ROS (red) and neutrophils (green) of LNVs pre-treated larvae resulted significantly (p value < 0.5) less abundant at the inflammation site in comparison to ctrl larvae (Figures 5C and 5D). Moreover, when we considered the number of neutrophils actively producing ROS by counting the cells co-expressing red and green signals, the reduction of inflammatory response was even more evident (p value < 0.001) (Figure 5E).

**Figure 6:**
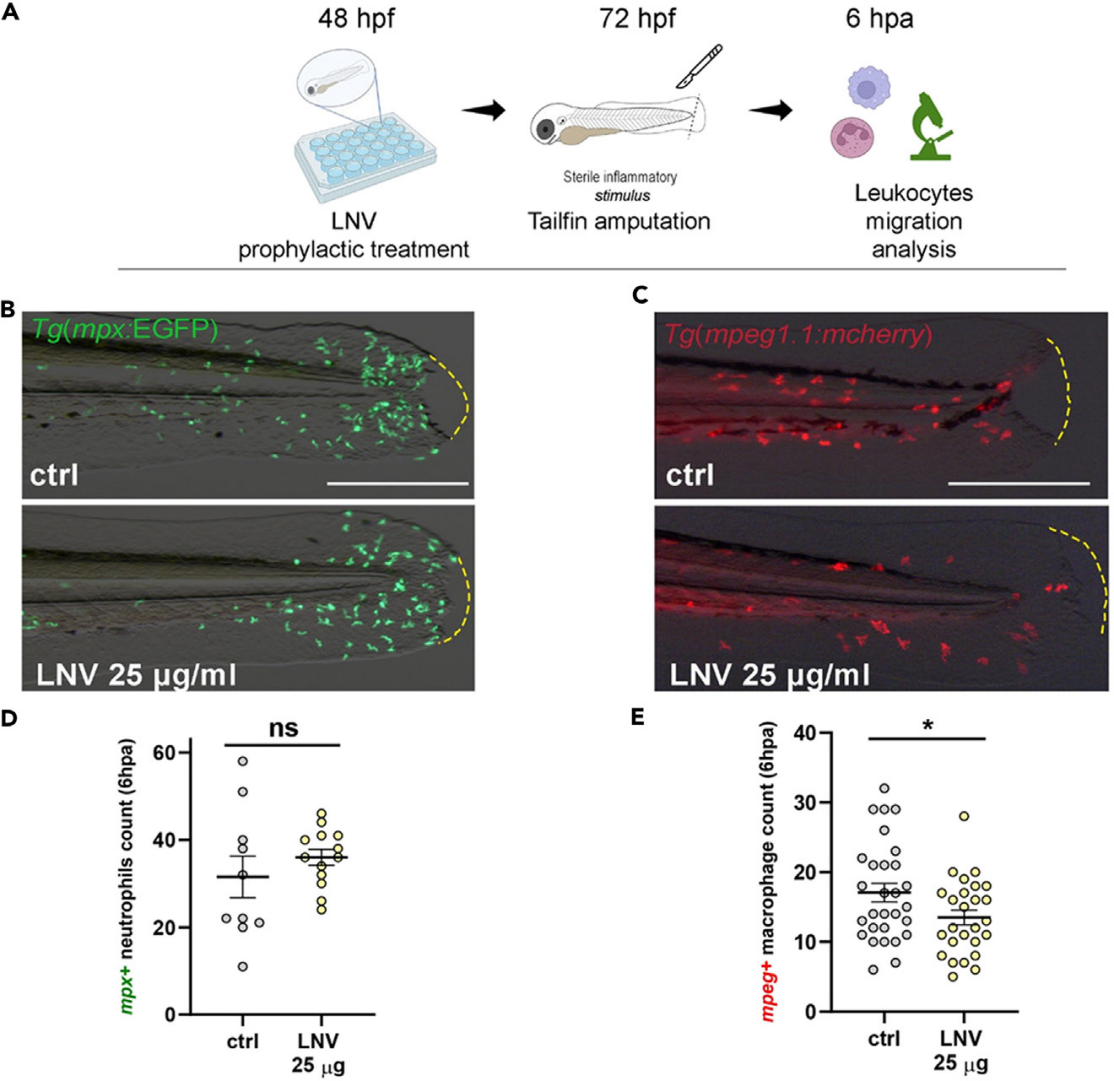
Anti-inflammatory effects of prophylactic treatment with LNVs on innate immunity of zebrafish embryo 48hpf embryos were prophylactically treated with 25 μg/mL of LNVs and then undergone to sterile local inflammatory stimulus. (A) Schematic representation of sterile inflammatory stimulus model; (B and C) Representative images of the trunk-tailfin region of wounded *Tg(mpx:GFP)* (B) and *Tg(mpeg1.1:mcherry)* (C) embryos at 6 hpa, treated or not with LNV; (D and E) quantitative analysis (fluorescence cell count) of neutrophils (D) or macrophages (E) recruitment at the site of tailfin amputation. Mean and SEM of at least two independent experiments are shown; dots represent cell count in a single embryo. Statistical significance was assessed by unpaired Student’s *t* test followed by Welch’s correction (the Gaussian data distribution were assessed by Kolmogorov-Smirnov normality test). ∗∗∗p < 0.05; ∗∗p < 0.01; ∗p < 0.05. Scale bar indicates 100 μm in panels B and C.

To assess if the prophylactic administration of LNVs induces an effect on the modulation of the innate immune system, we took advantage of the reporter lines for neutrophils and macrophages: the *Tg(mpx:GFP)* and *Tg(mpeg1.1:mcherry)* transgenic lines, respectively. Embryos were exposed to LNVs (25 μg/mL) from 48 to 72 hpf, and an acute inflammatory sterile stimulus was induced by cutting a small portion of larvae tailfin without damaging the circulatory loop as previously described.^42^ Larvae were incubated at 28°C for 6 h post-tailfin amputation (6 hpa), to reach the peak of neutrophils recruitment at the wound site^43^ (Figure 6A). We observed that the number of mcherry + macrophages recruited at the wound was significantly reduced in LNVs-treated larvae in comparison to controls (ctrl) (Figure 6C). Indeed, while ctrl larvae presented a mean of about 17 mcherry + macrophages at the wound site per embryo, in LNVs-treated larvae the number decreased to about 13.5 per embryo (Figure 6E). No differences have been observed in neutrophils recruitment at this stage of analysis (Figures 6B–6D).

We performed also experiments assessing the immunomodulatory potential of therapeutic treatment with LNVs, obtaining similar anti-inflammatory and antioxidant effects on zebrafish embryo (Figures S3 and S4).

## Discussion

Fruits and vegetables are rich in natural antioxidants, thus representing a good source of bioactive molecules. In particular, citrus fruits contain flavonoids, carotenoids, sugars, polyphenols, and dietary fiber.^44^ However, the chemical stability and bioavailability of most of the anti-inflammatory and antioxidant compounds contained in citrus fruit are strongly correlated with the storage and process conditions.^44^ Vitamin C (ascorbic acid), for instance, is a well-known antioxidant agent; however, its instability made its usage challenging.^45^

Plant-derived vesicles (PDEVs) represent naturally occurring nanoparticles that enclose and protect several bioactive metabolites, thus representing attractive therapeutic tools.^46^ The number of studies focused on the beneficial effects of PDEVs has grown exponentially in the last few years. Increasing evidence has shown the variety of beneficial effects played by PDEVs in cross-kingdom communication^2^; in particular, it was found that PDEVs can inhibit tumor growth^6^^,^^47^ and reduce inflammation.^7^^,^^48^ Previous works have shown the antioxidant properties of PDEVs; in particular, those isolated from strawberries,^29^ carrots,^15^ and blueberries.^28^ Moreover, a very recent study demonstrated that EVs isolated from a medical fungus, *Phellinus linteus*, possessed fascinating antioxidant and anti-aging properties on human skin cells.^49^ Here, we investigated the possible antioxidant properties of nanovesicles isolated from lemon juice (LNVs) using *in vitro* and *in vivo* models.

Although, we have already performed a deep characterization of LNVs in our previous studies,^6^^,^^7^ here we further characterized LNVs according to MISEV guidelines.^30^ Thanks to AFM we could confirm the average size of LNVs (80 nm), which is in concordance with our previous observations.^6^ Moreover, we detected the presence of HSP70, a well-known EV marker in mammalian ones,^50^ thus confirming that LNVs can be considered EV-like nanoparticles. Considering the need for standardization for EVs isolated from plant matrices^51^ we cannot exclude that our LNVs could be a mixture of both extracellular and intracellular vesicles. However, the possibility to isolate LNVs from *Citrus limon* juice, with a high yield, and their demonstrated beneficial properties, prompted us to deeply and further evaluate their *in vitro* and *in vivo* effects.

Once we isolated and characterized LNVs, we tested their effects on the viability in *in vitro* and *in vivo* models, represented by human dermal fibroblast and zebrafish embryos. First, we assessed that LNVs did not impair the viability of human dermal fibroblasts (Figure 1D); this is in concordance with our previous results obtained in other normal cell lines.^7^ Moreover, the safety of LNVs was also confirmed *in vivo*: exposure to different doses of LNVs did not affect the morphology of 72 hpf zebrafish embryos and did not increase embryo mortality (Figure S1C). The biocompatibility of LNVs makes them attractive for possible therapeutic applications.

Reactive oxygen species production plays a key role in different physiological processes since they act as signaling molecules in mediating cellular proliferation, differentiation, and function.^52^

However, increasing studies demonstrated that excess in ROS production leads to oxidative stress and cellular damage. Oxidative stress is a pathological condition caused by an excess in ROS species which can alter lipids, proteins, and nucleic acids and lead to apoptosis.^17^ Natural compounds are enriched in antioxidant molecules thus representing valuable resources to contrast oxidative stress; moreover, PDEVs possess antioxidant properties.^15^^,^^28^^,^^29^ However, up to our knowledge, the antioxidant properties of PDEVs were evaluated just *in vitro*.

In this work, we demonstrated that LNVs could enhance the physiological functions of human dermal fibroblasts, inducing the production of ECM proteins, such as COLA1α1 and hyaluronic acid (HA), decreasing the level of COX-2, an enzyme critical for prostaglandin biosynthesis. Moreover, LNVs prevent the production of ROS induced by two known oxidative stress stimuli:H_2_O_2_ and UVB irradiation. The inhibition of ROS production may be mediated by different compounds found in PDEVs; in a recent study, it has been demonstrated that organic agriculture-derived nanovesicles contain catalase, superoxide dismutase 1 (SOD-1), glutathione (GSH), and ascorbic acid, with known antioxidant properties.^53^ In our previous study, we characterized LNV content and identified several flavonoids and limonoids.^7^ Among them, hesperidin, vicenin-2, and eriocitrin have antioxidant activities both *in vitro* and *in vivo,*^54^^,^^55^^,^^56^ which could explain the obtained results. Duan et al. demonstrated that vicenin-2 exert a protective effects against oxidative stress in human dermal fibroblasts stimulated with UVB irradiation.^55^ Another study showed that hesperidin exerts antioxidant effects *in vivo* playing a protective effect against cardiotoxicity.^57^

Recently, PDEVs from other sources showed antioxidant effects on other cell lines, such as mesenchymal stromal cells,^29^ hepatocellular carcinoma cells,^58^ cardiomyoblast, and neuroblastoma cells.^15^ Moreover, it was demonstrated that *Aloe Saponaria*^59^ and *Aloe vera*-derived EVs^60^ increase the migration of dermal fibroblasts.

Our finding showed that LNVs can activate AhR/Nrf2 signaling pathway in human dermal fibroblasts both under physiological and oxidative conditions. AhR activation can exert protective effects toward oxidative stress by activating the Nrf2 transcription factor. Recent evidence highlighted that many antioxidant phytochemicals can simultaneously activate AhR and Nrf2, thus leading to an antioxidant response.^41^ Besides, it is known that Nrf2 can be activated by several plant extracts and promotes skin tissue regeneration.^61^ Although, it was previously assumed that Nrf2 plays a role just in oxidative stress response, now it is well known that the activation of Nfr2 also mediates anti-inflammatory effects.^62^^,^^63^ Kobayashi et al. found that Nrf2 inhibits LPS-induced expression of pro-inflammatory cytokines binding interleukin 6 and interleukin 1b loci.^64^ Previous studies demonstrated that some compounds identified in LNVs are able to activate AhR/Nrf2 pathway. He et al. found that eriocitrin alleviates oxidative stress *in vivo* by activating Nfr2/NQO-1/HO-1/NF-kB proteins.^56^ Another group found that hesperidin counteracts oxidative stress through the activation of Nrf2 pathway in a mice model of acute renal damage.^65^ It has been also demonstrated that quercetin can bind and activate AhR in hepatic cells.^66^ For the aforementioned reasons, we focused our attention on this signaling pathway. We observed an increase in the protein level of AhR in cells cultured with LNVs and in its nuclear translocation when LNV-treated cells were stimulated with UVB. At the same time, Nrf2 was upregulated in human dermal fibroblasts treated with LNVs, and its nuclear localization was increased after the UVB stimulation. The activation of the AhR/Nrf2 signaling pathway can explain the reduction of ROS mediated by LNVs in human dermal fibroblasts stimulated with both H_2_O_2_ and UVB irradiation. While Nrf2 is a well-known mediator of the antioxidant response,^67^ AhR can be activated by both oxidative ligands and antioxidant phytochemicals.^41^ Moreover, AhR represent an environmental sensor that can be activated also following UV irradiation, as demonstrated in another study by Gao et al.^27^ The activation of AhR alone may lead to an increase of ROS production, while when AhR activates Nrf2 it takes part to the antioxidant response.^27^^,^^68^ Moreover, the LNV-mediated increase in cell migration, both without and with oxidative stress stimulus, may be correlated with Nfr2 up-regulation. The activation of the AhR/Nrf2 pathway is mediated by several phytochemicals enclosed in LNVs, which we analyzed in our previous work^7^; however, the most plausible possibility is that underlying this mechanism is not a single compound but multiple molecules that carried by LNV may act together and synergistically. Finally, we cannot exclude that the observed antioxidant effects may, at the same time, be mediated by the activation of other signaling pathways, which take part to the oxidative stress response.

The new field of natural products is in expansion and needs the use of models to evaluate the effects of metabolites, including the determination of the maximum tolerable dose. Several animal models are used *in vivo*, and among them, zebrafish embryos have gained interest in recent years because of their rapid reproduction, skin transparency, easy manipulation, and gene conservation with humans. The small size of zebrafish embryos that easily fit in a 96-well plate, makes them suitable for metabolites testing as the quantities of compounds required are minimal and costs are reduced. Recent works used this valuable model for the study of oxidative stress-linked disorders, focusing on the biological activities of natural compounds^69^^,^^70^^,^^71^

Zebrafish is a model to study the toxicity and biocompatibility of several compounds.^72^ In a study by Eissa et al. it was demonstrated that zebrafish embryos stimulated with LPS could represent a good model to study the anti-inflammatory effects of *Aquilaria malaccensis* leaf extract.^73^

To our knowledge, this is the first study in which plant-derived EVs’ effects were evaluated in this animal model. Here, we confirmed the antioxidant effects of LNVs by using two different models of oxidative stress induction in zebrafish embryos, a sterile inflammation,^74^^,^^75^^,^^76^ and an LPS-induced inflammation.^77^^,^^78^ Notably, we performed the experiments at a developmental time point in which the innate immune system differentiated with active neutrophils and macrophages.^79^ Our results are in line with those from other research groups that highlighted the robustness of zebrafish embryos model in the analysis of oxidative stress response, ROS production^80^ and the recruitment of innate immune cells.^81^ Future studies could investigate the effects of LNVs on IL-1β-mediated inflammation. Indeed, by taking advantage of zebrafish embryos that share a conserved IL-1β structure with their human counterpart, anti-inflammatory effects of LNVs can be monitored following specific IL-1β expression in leukocyte. IL-1β is detectable only following injury and might be a clear readout of the inflammatory process.^82^

**Figure 7:**
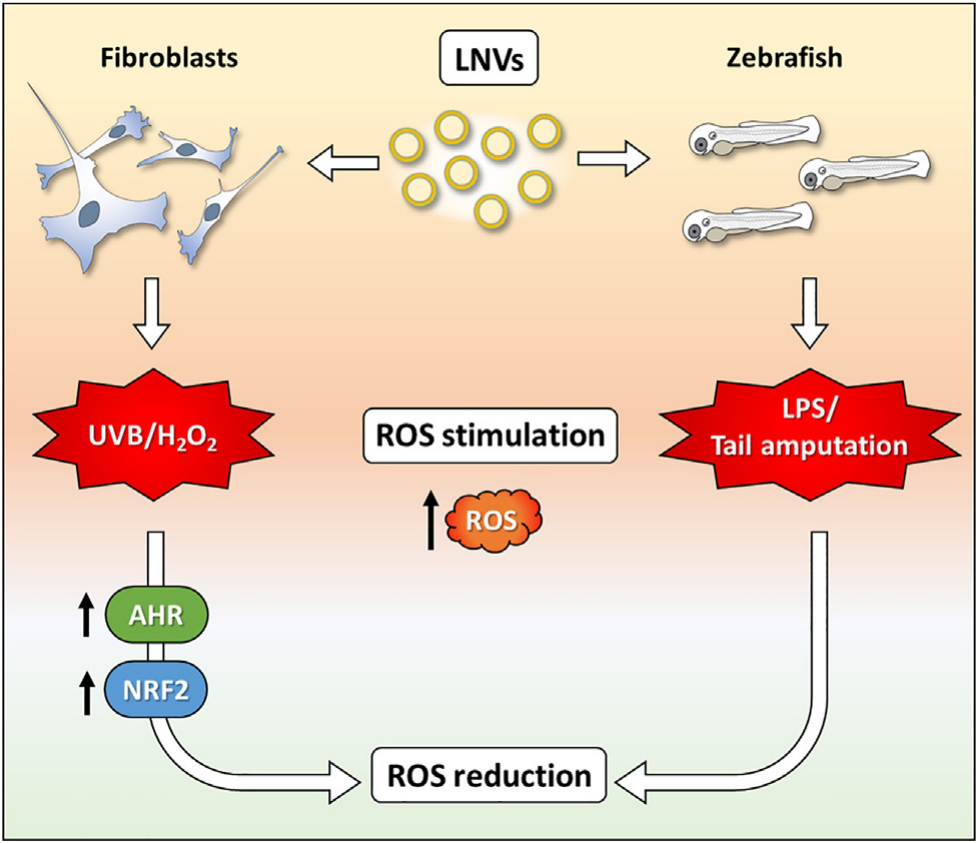
Visual summary of the main findings of the article

To conclude, here for the first time we demonstrated the antioxidant and anti-inflammatory properties of PDEVs isolated from *Citrus limon* juice both *in vitro*, using human dermal fibroblasts, and *in vivo*, in zebrafish embryos. Our findings showed the ability of LNVs to decrease ROS species in fibroblasts stimulated with H_2_O_2_ and UVB irradiation. These results were confirmed in zebrafish, emphasizing the biocompatibility of LNVs and further validating their antioxidant and anti-inflammatory effects (Figure 7). Although additional studies are needed to better understand the underlying mechanism of LNVs action, this study encourages the clinical translation of PDEVs for the prophylactic treatment of oxidative stress-mediated pathological conditions.

### Limitations of the study

In this study, we observed that LNVs exert antioxidant effects by decreasing ROS production both *in vitro* and *in vivo*. Although the preliminary results show a correlation between ROS decrease and the AhR-Nrf2 signaling pathway, more comprehensive, and detailed *in vivo* experiments are needed to confirm and deeply investigate the involvement of this mechanism in the LNV-mediated ROS decrease.

## STAR★Methods

### Key resources table

**Table:** 

REAGENT or RESOURCE	SOURCE	IDENTIFIER
**Antibodies**		

anti-HSP70 antibody	Agrisera	Cat# AS08 371; RRID: AB_2295493
anti-AhR antibody	Novus Biologicals	Cat# NB100-128SS; RRID: AB_10011093
anti-Nrf2 antibody	Novus Biologicals	Cat# NBP1-32822; RRID: AB_10003994
anti-Tubulin antibody	SantaCruz Biotechnology	Cat# sc-5286; RRID: AB_628411
Goat Anti-Mouse IgG, Light-Chain Specific Antibody (HRP Conjugate)	Cell signaling	Cat# 91196; RRID: AB_2940774
Goat anti-Rabbit IgG (H+L) Secondary Antibody, HRP	Invitrogen	Cat# 31460; RRID: AB_228341
Goat anti-Rabbit IgG Secondary Antibody, DyLight 594	Invitrogen	Cat# 35560; RRID: AB_1185570
Goat anti-Rabbit IgG Secondary Antibody, DyLight 488	Invitrogen	Cat# 35552; RRID: AB_844398

**Chemicals, peptides, and recombinant proteins**		

DCFH-DA	Sigma Aldrich	Cat# 35845
Fibroblast Basal media	ATCC	Cat# PCS-201-030
Fibroblast Growth Kit-Low serum	ATCC	Cat# PCS-201-041
Penicillin-Streptomycin Solution 100X	Euroclone	Cat# ECB3001
PBS	Euroclone	Cat# ECB4053
E3 fish growth medium	Instant Ocean	
1-phenyl-2-thiourea	Sigma Aldrich	Cat# 189235
Ethyl 3- aminobenzoate methanesulfonate salt	Sigma Aldrich	Cat# E10521
MTT (3-(4,5-Dimethylthiazol-2-yl)-2,5-Diphenyltetrazolium Bromide)	Invitrogen	Cat# M6494
Paraformaldehyd	Sigma Aldrich	Cat# 30525-89-4
TritonX-100	Sigma Aldrich	Cat# 9036-19-5
PowerUp™ SYBR™ Green Master Mix	Invitrogen	Cat# A25742

**Critical commercial assays**		

Pierce™ Coomassie Plus (Bradford) Assay Kit	Thermo Scientific™	Cat# 23236
RealTime-Glo™ MT Cell Viability Assay	Promega	Cat# G9711
IllustraTM RNA spin mini-RNA isolation Kit	GE Healthcare	Cat# 25-0500-72
High-Capacity cDNA Reverse Transcription kit	Applied Biosystems	Cat# 4368814
PKH26 Red Fluorescent Cell Linker Kits	Merck KGaA	Cat# PKH26GL-1KT
Dihydroethidium	SantaCruz biotechnology	Cat# 104821-25-2

**Experimental models: Cell lines**		

HDFα	ATCC	Cat# PCS-201-012, lot number 70017799

**Experimental models: Organisms/strains**		

Zebrafish (*Danio rerio*)	Zebrafish facility, University of Milan, Via Celoria, 26, 20133, Milan Auth. 198283 2019 D.Lgs 26/2014	https://zfin.org/action/feature/wildtype-list
transgenic reporter line Tg(mpx:GFP)	Zebrafish facility, University of Milan, Via Celoria, 26, 20133, Milan Auth. 198283 2019 D.Lgs 26/2014	https://zfin.org/ZDB-TGCONSTRCT-120906-1
transgenic reporter line Tg(mpeg1.1: mcherry)	Zebrafish facility, University of Milan, Via Celoria, 26, 20133, Milan Auth. 198283 2019 D.Lgs 26/2014	https://zfin.org/ZDB-TGCONSTRCT-120117-2

**Oligonucleotides**		

Primers see table in real-time PCR	N/A	N/A

**Software and algorithms**		

Prism (GraphPad Software)	GraphPad	https://www.graphpad.com/
ImageJ Software	ImageJ	https://imagej.net/ij/
Image lab	Bio-Rad	https://www.bio-rad.com/it-it/product/image-lab-software?ID=KRE6P5E8Z

**Other**		

Actin Green	Molecular Probes	Cat# R37110
Hoechst	Molecular Probes, Life Technologies, Carlsbad, CA, USA	Cat# H3570
UV lamp	CAMAG	Cat# 29230
*Pseudomonas aeruginosa* LPS	ATCC	Cat# 27316

### Resource availability

#### Lead contact

Further information and requests for resources and reagents should be directed to and will be fulfilled by the lead contact, Stefania Raimondo (stefania.raimondo@unipa.it).

#### Materials availability

This study did not generate new unique reagents.

### Experimental model and study participant details

#### HDFα cell line

The human dermal fibroblast HDFα cell line was purchased from ATCC (Manassas, VA, USA). Cells were cultured in Fibroblast Basal media (ATCC, Manassas, VA, USA) supplemented with Fibroblast Growth Kit-Low serum (ATCC, Manassas, VA, USA), 100 U/ml penicillin, and 100 μg/ml streptomycin (Euroclone, UK). HDFα cells were grown in a 37°C humidified incubator with 5% CO_2_ and were passaged every 2-3 days. The donor gender of the cell line is male.

#### Zebrafish

Zebrafish (*Danio rerio*) were maintained at the University of Milan, Via Celoria 26 – 20133 Milan, Italy (Aut. Prot. n. 295/2012-A – December 20, 2012) according to international (EU Directive 2010/63/EU) and national guidelines (Italian decree No. 26 of the 4th of March 2014). Embryos were collected by natural spawning, staged according to Kimmel et al. 32, and raised at 28.5°C in E3 fish growth medium (Instant Ocean, 0.1% Methylene Blue) in Petri dishes, according to established techniques. After 24h post fertilization (hpf), 0,003% 1-phenyl-2-thiourea (PTU, Sigma Aldrich) was added to the fish water to prevent pigmentation. Embryos were washed, dechorionated, and anesthetized with 0.016% tricaine (Ethyl 3- aminobenzoate methanesulfonate salt; Sigma-Aldrich), before observations, microinjection, and image acquisitions.

### Method details

#### Lemon nanovesicle (LNV) isolation

Nanovesicles were isolated from *Citrus limon L.* juice as previously described.^6^ Fruits were obtained from a private farmer, carefully washed in water, and manually squeezed. The juice was sequentially centrifuged at 3,000 × g for 15 minutes, and 10,000 × g for 1 hour. The supernatant was filtered at 0.8 μm and 0.45 μm pore filter and centrifuged at 16,500 × g for 3 hours. Then the supernatant was ultra-centrifuged at 100,000 × g for 105 minutes in a Type 70 Ti, fixed angle rotor, the pellet was washed and suspended in phosphate-buffered saline (PBS). LNVs quantification was determined with the Bradford assay (Pierce, Rockford, IL, USA). On average, from 240 ml of *Citrus* juice, we recovered 600 micrograms of vesicles suspended in PBS, which correspond to 1.87x10^11^ particles (3.13x10^11^ particles/ml).

#### Atomic force microscope

A 30 μl LNVs solution, diluted in PBS to a final concentration of 1 μg/ml, was deposited onto freshly cleaved mica for 15 minutes at room temperature. After a gentle rinsing by PBS, AFM measurements were carried out in the same buffer by a Nanowizard III scanning probe microscope (JPK Instruments AG, Germany) equipped with a 15 μm z-range scanner. Different size images were acquired in Quantitative Imaging mode by using an AC40 (Bruker) cantilever (spring constant 0.15 N/m, calibrated by thermal method and JPK software,^83^ typical tip radius 10 nm) and setting force setpoint 150 pN, Z-length 50 nm, pixel time 5 ms.

#### Nanoparticle Tracking Analysis

Particles size distribution and concentration were measured by Nanoparticle Tracking Analysis (NTA) (NanoSight NS300, Malvern Instruments Ldt, UK). To get the ideal concentration for instrument linearity, the samples were diluted 1:100 in PBS.

#### Western blotting

Total proteins from LNVs and HDFα cells treated with LNVs (10 and 25 μg/ml) or pre-treated with LNVs and then exposed to UVB irradiation (20 mJ/cm^2^) for 25 seconds were isolated and analyzed by SDS-PAGE followed by western blotting. Antibodies used in the experiments were as follows: anti-HSP70 antibody (Agrisera, Vännäs, Sweden), anti-AhR antibody (Novus Biologicals, Milano, Italy), anti-Nrf2 antibody (Novus Biologicals, Milano, Italy), and anti-Tubulin antibody (Santa Cruz Biotechnology, Heidelberg Germany). The membranes were incubated with HRP-conjugated secondary antibody (Thermo Fisher Scientific, Cambridge, MA, USA) and the chemiluminescent signal was detected by Chemidoc (Biorad, Milan, Italy).

#### Confocal microscopy

LNVs were labeled with PKH26 Red Fluorescent Cell Linker Kits (Merck KGaA, Darmstadt, Germany) following the datasheet information. Briefly, LNVs were incubated with PKH26 dye for 15 min at room temperature, washed twice in PBS, and resuspended in the growth medium. The labeled LNVs were incubated with HDFα cells for 4 h at 37°C with 5% CO_2_ and at 4°C. After incubation, the cells were fixed with PFA 4%, permeabilized with 0.1% TritonX-100, and stained with Actin Green (Molecular Probes, Life Technologies, Carlsbad, CA, USA) and Hoechst (Molecular Probes, Life Technologies, Carlsbad, CA, USA).

HDFα were treated with LNVs (10 and 25 μg/ml) for 24h, then the cells were fixed with PFA 4%, permeabilized with 0.1% TritonX-100, incubated with anti-AhR antibody (Novus Biologicals, Milano, Italy) or anti-Nrf2 antibody (Novus Biologicals, Milano, Italy) for 1h, then washed and incubated with Goat anti-Rabbit IgG Secondary Antibody, DyLight 594 or 488 (Invitrogen) for 1h. Actin was stained using Actin Green (Molecular Probes, Life Technologies, Carlsbad, CA, USA), and nuclei were stained with Hoechst (Molecular Probes, Life Technologies, Carlsbad, CA, USA).

The samples were analyzed by confocal microscopy (Nikon A1, Amsterdam, Netherlands).

#### Cell viability assays

Cell viability was determined by 3-[4,5-Dimethylthiazol-2-yl]-2,5 Diphenyl Tetrazolium Bromide (MTT) assay and RealTime-Glo™ MT Cell Viability Assay (Catalog number G9711, Promega, Madison, WI, USA). HDFα cells were seeded in triplicate in 48-well plates; 24 h post-seeding, cells were treated with different doses of LNVs (10, 25, and 50 μg/ml) for 24 and 48h.

For the *MTT assay*, the absorbance was measured by an ELISA reader at 540 nm (Microplate Reader, BioTek, Winooski, VT, USA). Values are expressed as a percentage of cell growth versus control (untreated cells).

For RealTime-Glo™ MT Cell Viability Assay (Catalog number G9711, Promega, Madison, WI, USA) HDFα cells were plated in triplicate into white-walled, opaque 96 well plates; 24h post-seeding, cells were treated with different doses of LNVs (10, 25, and 50 μg/ml). At the same time, the 2X MT Cell Viability Substrate and NanoLuc® Enzyme were added. The luminescent signal, which correlates with the number of metabolically active cells, was measured at 24, 48, and 72h by Glomax (Promega).

#### Real-time PCR

HDFα cells were seeded in 12 well-plates at 2×10^4^ cells/well; 24h post-seeding cells were treated with 10 or 25 μg/ml of LNVs for 24h. At the end of the treatments, total RNA was extracted using Illustra^TM^ RNA spin mini-RNA isolation Kit (GE Healthcare, Little Chalfont, Buckinghamshire, UK). The RNA was reverse transcribed to cDNA using the High-Capacity cDNA Reverse Transcription kit (Applied Biosystems, Foster City, CA, USA). Then, the cDNA was subjected to quantitative real-time reverse transcriptase-polymerase chain reaction (RT-PCR) analysis. The sequences of the primers used were listed in below table.

**Table:** 

Gene	Forward Sequence (5′to 3′)	Reverse Sequence (5′to 3′)
ACT	TCCCTTGCCATCCTAAAAAGCCACCC	CTGGGCCATTCTTCCTTAGAGAGAAG
COL1α1	TGTGGATGCCTCTTGGGTATC	TTTTGGCCATCTCTTCCTTCA
HAS2	GTCATGTACACAGCCTTCAGAGC	ACAGATGAGGCTGGGTCAAGCA
COX-2	CGGTGAAACTCTGGCTAGACAG	GCAAACCGTAGATGCTCAGGGA

Real-time PCR was performed using Step One^TM^ Real-time PCR System Thermal Cycling Block (Applied Biosystem) in a 20 μl reaction containing 300 nM of each primer, 2 μl template cDNA, 18 μl 2X SYBR Green I Master Mix. The PCR was run at 95°C for 20 sec followed by 40 cycles of 95°C for 3 sec and 60°C for 30 sec. Actin was used as the endogenous control. Relative changes in gene expression between control and treated samples were determined using the ΔΔCt method.

#### Oxidative stress induction *in vitro*

The oxidative stress was induced using both hydrogen peroxide (H_2_O_2_) and UVB irradiation. Briefly, HDFα cells were exposed to H_2_O_2_ (300 μM) for 4h or UVB irradiation (20 mJ/cm^2^) for 25 seconds. For UVB irradiation, the media was replaced with a tiny layer of PBS, the culture lid was opened, and the plates were placed under the UV lamp (CAMAG, cat n 29230, ser n 911136, V: 220, A: 0,2). After UVB irradiation, for ROS measurement, the experiment was stopped immediately, while for western blot analyses, the PBS was replaced with serum-free media, and cells were incubated for 24h, as previously described.^84^ Control cells were exposed to the same conditions, but without UV irradiation.

#### Wound healing assay

HDFα cells were seeded in 12 well-plates; once the cells reached 80-90% of confluence, they were treated with 10 or 25 μg/ml of LNVs for 24h. The day after, a “scratch” was generated with a p200 sterile pipet tip, and pictures of cells were acquired at 0, 3, and 6 h with an optical microscope (4X magnification). In the experiments with UVB irradiation, after the “scratch”, cells were irradiated with UVB as described in the previous paragraph. By using Image J, the surface area of the scratch was measured, and the percentage of wound closure was assessed with the formula WC % = [(A_t0_ - A_t1_)/A_t0_]x100, where A_t0_ is the surface area at t 0 (0h) and A_t1_ is the surface area at t 1 (3 or 6 h).

#### ROS measurement

The DCFH probe (Sigma Aldrich, Saint Louis, MO, United States) was used to detect the amount of ROS produced by HDFα cells. HDFα cells were plated in triplicate into white-walled, opaque 96 well plates; 24h post-seeding, cells were pre-treated with different doses of LNVs (10, and 25 μg/ml) and then exposed to H_2_O_2_ (300 μM for 4h or UVB irradiation (20 mJ/cm^2^) for 25 seconds. In cells stimulated with H_2_O_2_, the DCFH probe (20 μM) was added together with hydrogen peroxide, while in the UV-irradiated cells, the probe was added 30 minutes before the UV exposure. The fluorescent signal, which directly correlates with the amount of ROS produced by cells, was immediately measured by Glomax (Promega).

#### LNV prophylactic treatment of zebrafish larvae

For prophylactic treatment, 48 hpf larvae were used. Larvae were divided into groups of 10 and each group was transferred into a 24-well plate with a total volume of 1 ml of E3 + PTU + LNV suspension at a final concentration of 10, 25 or 50 μg/ml per well. Control embryos were kept in E3 + PTU medium without LNVs. Larvae were incubated at 28.5°C for 24h in the dark, to avoid the degradation of the photosensitive LNVs.

#### Acute inflammatory stimulus in zebrafish larvae

To study leukocytes activation, *TgBAC(mpx:EGFP)i114*^74^ *(Tg(mpx:GFP))* and *Tg(mpeg1.1:mcherry)*^85^ transgenic reporter lines were used to follow the behaviour of neutrophils and macrophages, respectively. A model of acute inflammation in zebrafish larvae was generated through two different types of inflammatory stimuli: via an intramuscular microinjection of lipopolysaccharide (LPS) of *Pseudomonas aeruginosa* (*Pa*) (*Pa*-LPS) (derived from strain ATCC 27316, Sigma Aldrich) and via a sterile inflammation, by amputation of the tailfin. For local LPS inflammatory stimulus, larvae at 72 hpf were microinjected with 1 nl of pure *Pa-*LPS suspension into the skeletal muscle of the trunk region as described in, by delivering the suspension in the region between the second and the fifth somite from the onset of the yolk extension. Embryos were incubated at 28.5°C in E3+PTU and leukocyte recruitment and ROS generation at the injection site were assessed after 4h post-amputation (hpa) by epifluorescence microscopy. For local sterile inflammatory stimulus, a portion of the tailfin of the embryo was amputated with a sterile scalpel blade, as described in. Amputated larvae were incubated at 28.5°C in E3+PTU and leukocyte recruitment in the tailfin area was assessed after 6 hpa. Single slice bright-field and fluorescence images were sequentially acquired using an epifluorescence stereomicroscope (M205FA, Leica, Wetzlar, Germany) equipped with a fluorescent lamp and a digital camera and mounting mcherry-filter (excitation 587 nm) and GFP-filter (excitation 488 nm). Macrophage and neutrophils recruitment at the wound site area was measured by counting *mcherry*^+^ or *mpx*^+^ cells in the defined region of interest, by computation using Fiji (Developer: Wayne Rasband), using “Find maxima” function, as described in Ellett and Lieschke.^86^ Adobe software was used to process the images.

#### Analysis of ROS generation in zebrafish larvae

*Tg(mpx:GFP)* larvae treated with LNVs from 48 hpf, stimulated by intramuscular microinjection of *P. aeruginosa-*LPS at 72 hpf, were assessed at 4 hpi for ROS production. The commercial kit DHE (dihydroethidium, Santa Cruz biotechnology, Dallas, Texas, USA) was used: 0.5 μl of 30 mM DHE stock solution was diluted in E3+PTU to obtain a final concentration of 5 μM. Larvae were exposed at 5 μM DHE for 15 minutes in the dark at 28.5°C, washed three times in E3 + PTU and immediately imaged using an epifluorescence stereomicroscope. ROS production was measured as red cells (ROS) related to green cells (neutrophils) as co-localization signal. Local neutrophil migration in the region of interest was measured through *mpx*^+^ cell count as described above. Computation analyses were done using Fiji as follows: different channels of images were merged, and brightness/contrast was adjusted for better visualization; a “color threshold” was set; “measure area” function was used to determine overlapped fluorescent pixels of the image to extrapolate *mpx*^+^DHE^+^ cell count.

### Quantification and statistical analysis

#### *In vitro* experiments

Data are reported as mean ± standard deviation (SD) of biological replicates. Statistical analysis was performed using GraphPad Prism software (GraphPad software, Inc, La Jolla, CA). The normal data distribution was assessed by Shapiro-Wilk test. When data follow normal distribution, the statistical significance of the differences was analyzed using a two-tailed Student’s t-test; otherwise, non-parametric method (Mann-Whitney test) were used to compare the groups. A p-value ≤0.05 was considered significant. The statistical details of each experiment can be found in the figure legends.

#### *In vivo* experiments

Statistical analyses were generated using GraphPad Prism software version 8.0.2 for Windows. The Gaussian data distribution of all datasets was guaranteed by the Shapiro-Wilk normality test or Kolmogorov-Smirnov normality test. Data resulted as outliers were excluded from the analysis. To evaluate the significance of differences between two groups, unpaired two-tailed Student’s t-test (followed by Welch’s correction when necessary) was used. As indicated in the relative figure legend, data represent the results of at least two independent experiments and mean ± SEM values were reported in graphs. P-value < 0.05 was considered to indicate statistically significant differences. The statistical details of each experiment can be found in the figure legends.

## Data Availability

• Catalog numbers are listed in the key resources table. • Original western blot images have been included as supplementary information figure. • Microscopy data reported in this paper will be shared by the lead contact upon request. • Any additional information required to reanalyze the data reported in this paper is available from the lead contact upon request.
