# Supplementary material for: Lemon-derived nanovesicles achieve antioxidant and anti-inflammatory effects activating the AhR/Nrf2 signaling pathway
Source: iScience. 2023 Jun 7;26(7):107041. doi: 10.1016/j.isci.2023.107041 (PMC10329147; doi:10.1016/j.isci.2023.107041)
Supplement: Document S1. Figures S1–S4 [file mmc1.pdf]

iScience, Volume *26*

Supplemental information

Lemon-derived nanovesicles achieve antioxidant and anti-inflammatory effects activating the AhR/Nrf2 signaling pathway

Ornella Urzì, Marco Cafora, Nima Rabienezhad Ganji, Vincenza Tinnirello, Roberta Gasparro, Samuele Raccosta, Mauro Manno, Anna Maria Corsale, Alice Conigliaro, Anna Pistocchi, Stefania Raimondo, and Riccardo Alessandro

Supplementary material

Supplementary figure 1

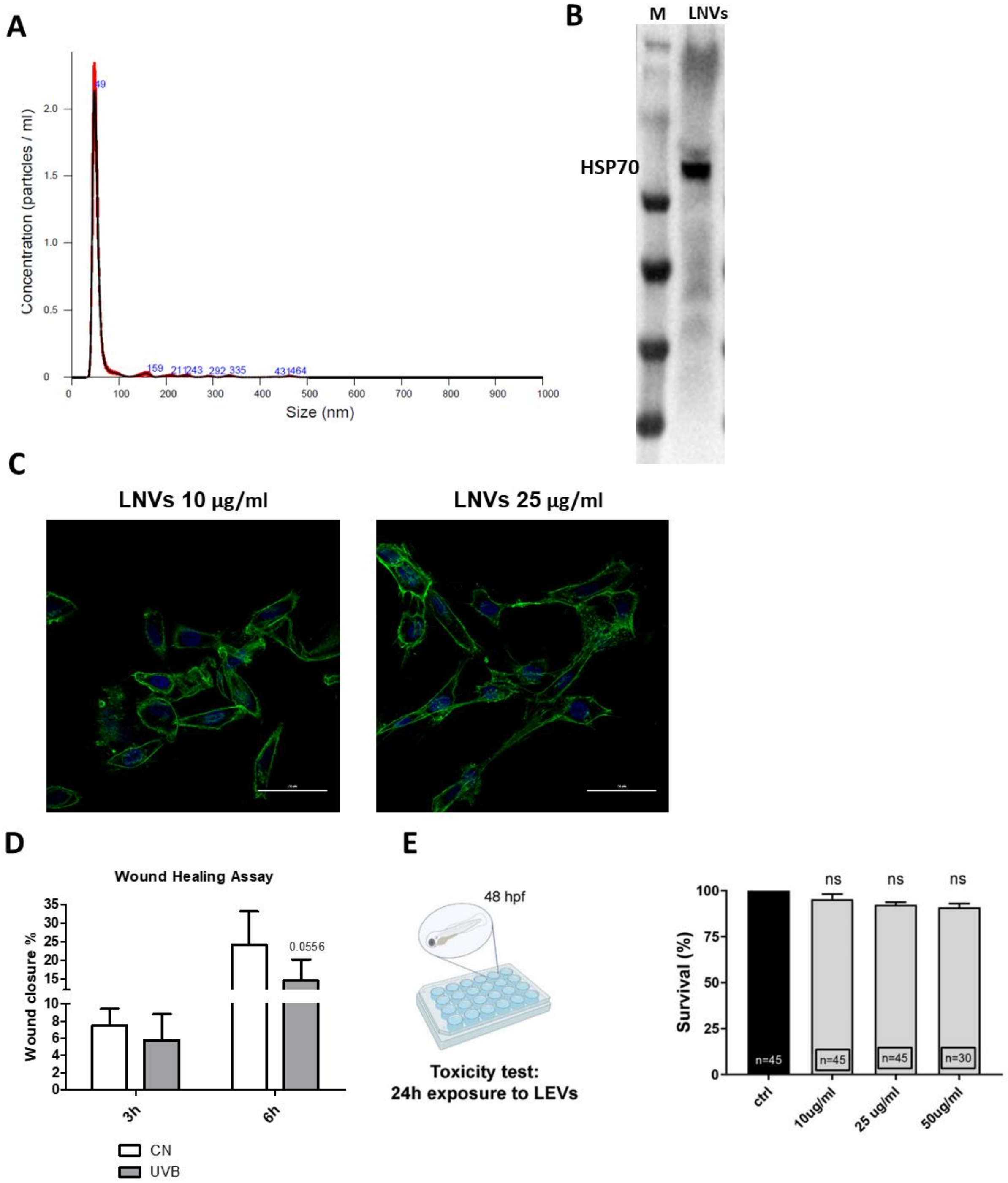

FIGURE S1 Characterization and uptake of LNVs by HDFα cells, related to Figure 1: (A) Size distribution of LNVs obtained through NTA. (B) Western blot uncropped membranes of results shown in Fig 1B. (C) LNVs internalization by HDFα cells at 4°C; LNVs were stained with PKH26 (in red), actin with Actin Green (in green), and nuclei with Hoechst (in blue). Scale bars are 50 µm. (D) **UVB exposure decreased the wound healing of HDFα cells, related to Figure 3:** Wound healing assay of HDFα cell line. After doing the scratch with a sterile 200 µl pipet tip, the cells were irradiated with UVB (20 mJ/cm^2) for 25 sec and observed at 0, 3, and 6 h. The percentage of wound closure was calculated as described in the Material and Methods section. Data are represented as the mean ±SD. (E) **Toxicity test of LNV on zebrafish embryos, related to Figure 5 and 6**. 48hpf embryos were exposed for 24 hours in a 24 well-plate to different concentrations of LNV suspension dissolved in E3+PTU. Mean values and SEM of the survival rate of treated embryos are reported on graphs. Data represent two independent experiments, and the number of embryos/doses is reported in the bars. Ordinary one-way ANOVA followed by post hoc Tukey's correction; ns=not significant.

Supplementary figure 2

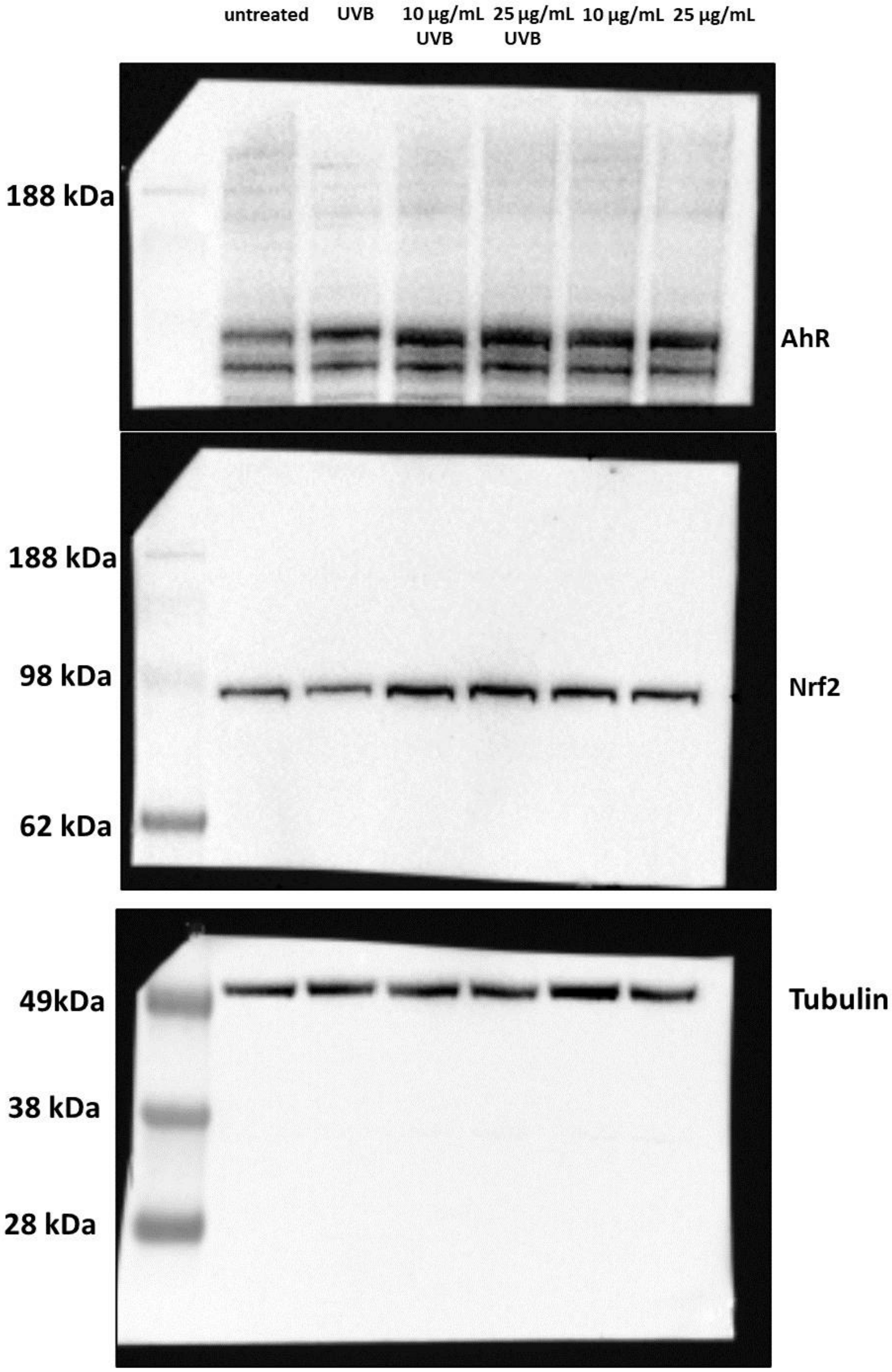

FIGURE S2 Western blot related to Figure 4A: uncropped membranes of results shown in Fig 4A.

Supplementary figure 3

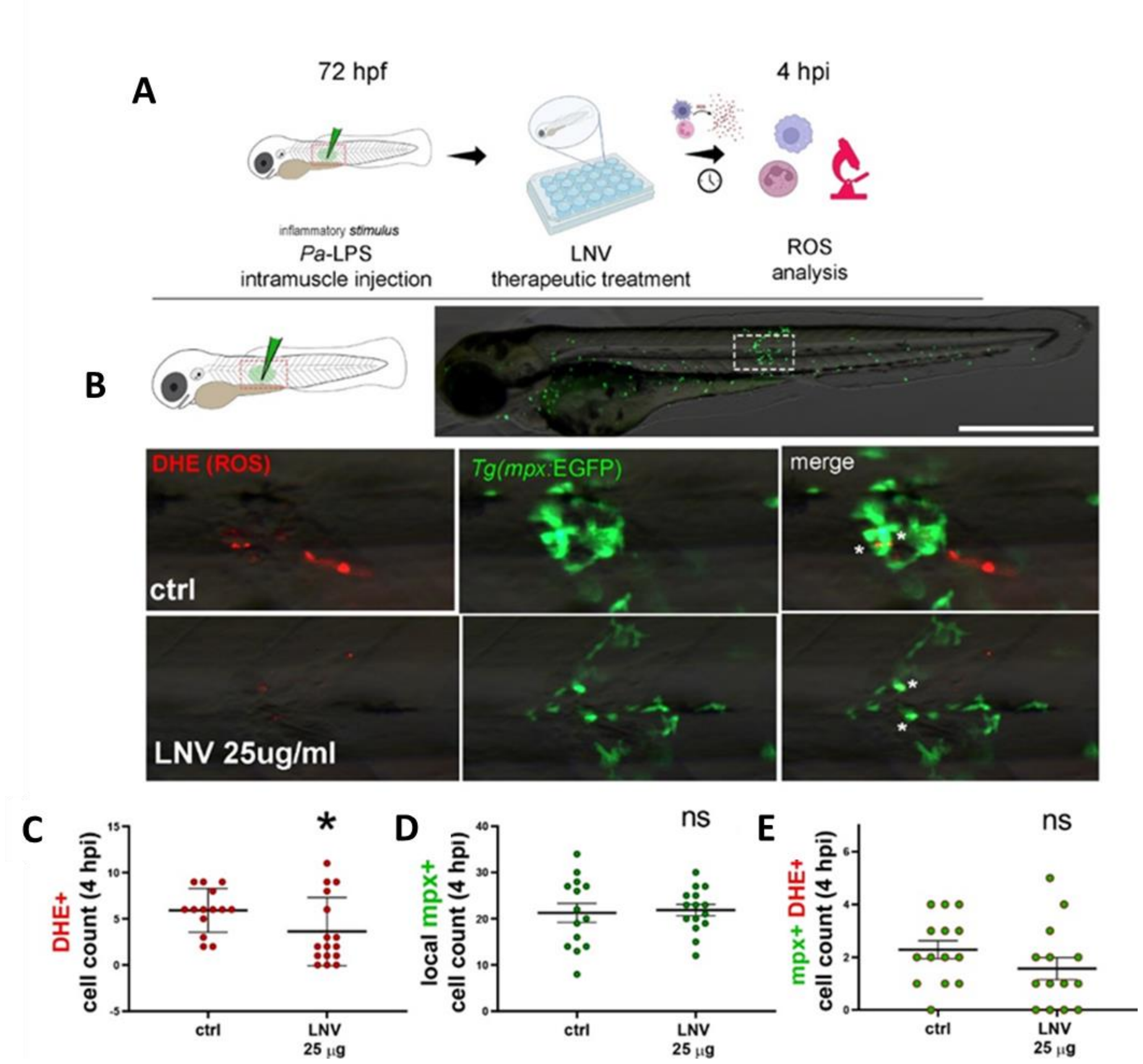

FIGURE S3 Antioxidant effects of therapeutic treatment with LNVs on innate immunity of zebrafish embryo, related to Figure 5. 72hpf embryos were stimulated with sterile Pa-LPS-induced local inflammatory stimulus and then treated with 10 or 25 µg/ml of LNVs. (A) Schematic representation of LPS-induced stimulus model; (B) representative images at 4 hpi of neutrophils recruitment and ROS production (DHE) at the site of Pa-LPS intramuscular injection, in the whole embryo (upper) and the region of interest of the trunk (lower panels), in embryos pre-treated or not with LNVs; white asterisks indicate mpx+DHE+ cells; (C-E) quantitative analysis at 4 hpi of DHE+ cell count (C), mpx+ cell count (D) and mpx+DHE+ cell count (E) at the region of interest. Mean and SEM of at least two independent experiments are shown; dots represent cell count in a single embryo. Statistical significance was assessed by unpaired Student's t test followed by Welch's correction (the gaussian data distribution was assessed by Kolmogorov-Smirnov normality test): ***p<0.05; **p < 0.01; *p < 0.05; ns not significant. The scale bar indicates 500 µm in panel B (upper) and 20 µm in panel B (lower).

Supplementary figure 4

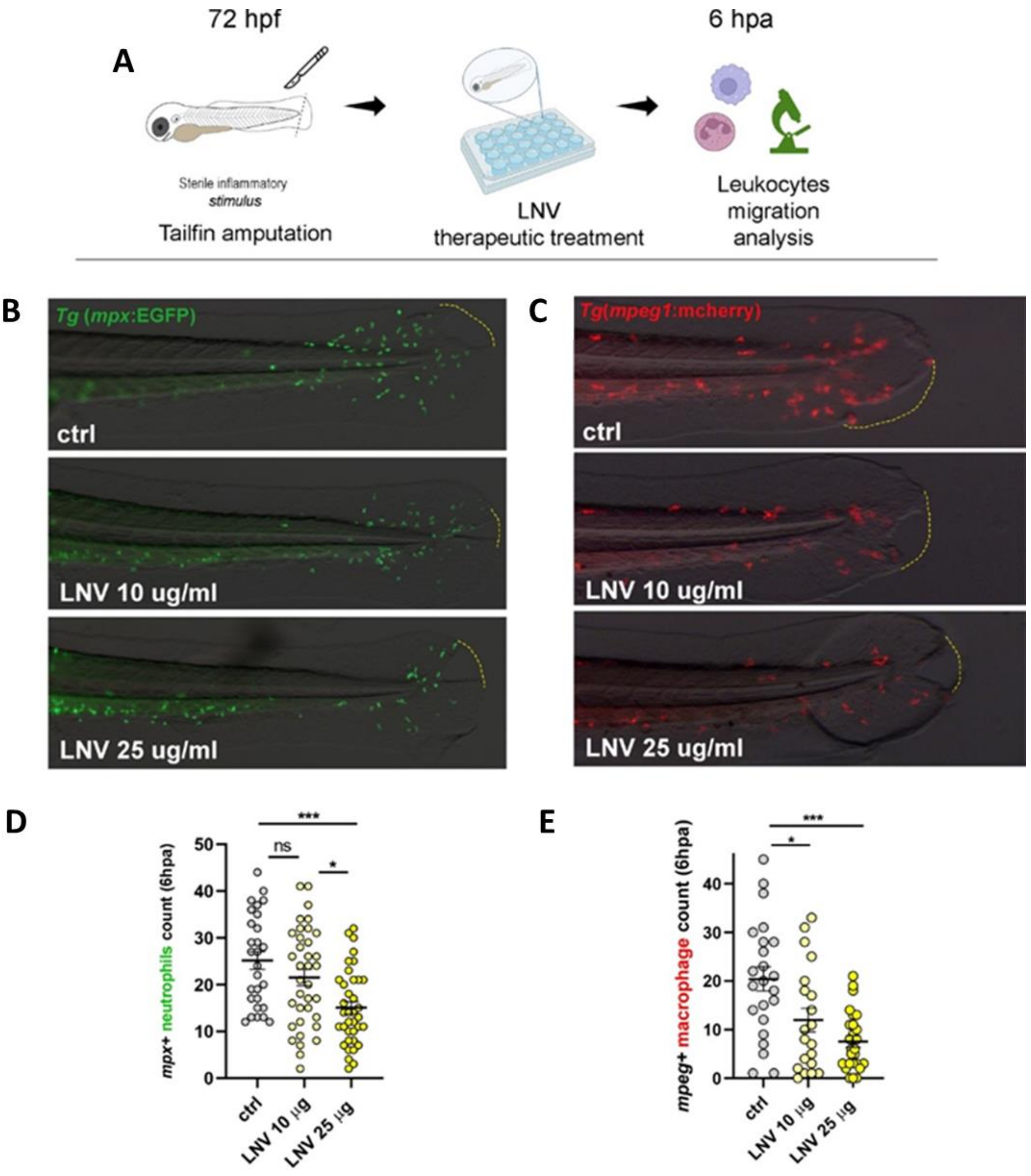

FIGURE S4 Anti-inflammatory effects of therapeutic treatment with LNVs on innate immunity of zebrafish embryo, related to Figure 6. 72hpf embryos were stimulated with sterile local inflammatory stimulus and then treated with 10 or 25 µg/ml of LNVs. A) Schematic representation of sterile inflammatory stimulus model; B-C) Representative images of the trunk-tailfin region of wounded Tg (mpx:GFP) (B) and Tg(mpeg1.1:mcherry) (C) embryos at 6 hpa, treated or not with different doses of LNV; (D-E) quantitative analysis (fluorescence cell count) of neutrophils (D) or macrophages (E) recruitment at the site of tailfin amputation. Mean and SEM of at least two independent experiments are shown; dots represent cell count in a single embryo. Statistical significance was assessed by one-way ANOVA followed

by Tukey's post hoc (the gaussian data distribution was assessed by Kolmogorov-Smirnov normality test): ***$p<0.05$; **$p < 0.01$; *$p < 0.05$. Scale bar indicates 100 μm in panels B and C.
